# Potential oxidative stress related targets of mitochondria-focused therapy of PTSD

**DOI:** 10.3389/fphys.2023.1266575

**Published:** 2023-11-13

**Authors:** Hanna Kmita, Graziano Pinna, Volodymyr I. Lushchak

**Affiliations:** ^1^ Department of Bioenergetics, Institute of Molecular Biology and Biotechnology, Faculty of Biology, Adam Mickiewicz University, Poznań, Poland; ^2^ Psychiatric Institute (SPHPI), Chicago, IL, United States; ^3^ UI Center on Depression and Resilience (UICDR), Chicago, IL, United States; ^4^ Center for Alcohol Research in Epigenetics (CARE), Department of Psychiatry, College of Medicine, University of Illinois at Chicago, Chicago, IL, United States; ^5^ Department of Biochemistry and Biotechnology, Vasyl Stefanyk Precarpathian National University, Ivano-Frankivsk, Ukraine; ^6^ Research and Development University, Ivano-Frankivsk, Ukraine

**Keywords:** PTSD, mitochondria, reactive oxygen species, monoamine oxidase, mitochondria-derived peptides, mitochondria-derived vesicles

## Abstract

Post-traumatic stress disorder (PTSD) remains a highly prevalent, under-diagnosed, and under-treated psychiatric disorder that often deteriorates over time, and is highly comorbid with major depressive disorder, suicidality, and substance use disorder. Several biomarkers have been proposed but have yet to be implemented into clinical practice. Treatments, including selective serotonin reuptake inhibitors, are efficacious in only a small number of patients, which underscores the need to develop novel, efficient treatments. Mitochondrial dysfunction resulting from chronic oxidative stress has been linked with both altered neurotransmitter signaling and the inflammatory response. Hereinafter, we discuss mechanisms by which mitochondrial dysfunction may contribute to the development of PTSD symptoms, and how these may even increase PTSD susceptibility. We also highlight possible therapeutic targets to reduce oxidative stress to prevent or treat PTSD symptoms.

## 1 Introduction

The pathophysiology of post-traumatic stress disorder (PTSD) is highly complex, and PTSD remains a largely underdiagnosed and undertreated mood disorder (Blakey et al., 2022). The diversity of factors that can precipitate PTSD symptoms, including acute stressors or the repetition of several traumatic events, often during childhood, complicates the therapeutic management of this highly debilitating psychiatric disorder (Yehuda et al., 2015). Causes for developing PTSD vary from childhood abuse to natural disasters (tsunami, earthquakes), and may include rape, participation in warzone combat situations, or being exposed to domestic violence as a mother or child. Symptoms, such as re-experiencing, nightmares, flashbacks, and an exaggerated fear response, generally persist for longer than a month after stress exposure and, when not properly treated with psychotherapy or pharmacotherapeutic approaches, may further worsen into chronic PTSD (Miao et al., 2018). Chronic PTSD exhibits high comorbidity with major depressive disorder and often, PTSD subjects also develop suicidal ideation and attempts (Muresanu et al., 2022).

First-line pharmacologic treatments include selective serotonin reuptake inhibitors (i.e., sertraline, paroxetine), which are the only FDA-approved drugs for this condition (Martin et al., 2021). Psychotherapy in the form of prolonged exposure therapy is also common in clinical practice. Regrettably, these therapeutic approaches only help a small portion of clients seeking treatment, and often show large non-response and relapse rates (Merians et al., 2023).

The lack of reliable biomarkers makes diagnosis, as well as developing more efficacious treatments, difficult (Aspesi and Pinna, 2018; Pinna, 2019). PTSD animal models are useful, but they only minimally reproduce the neurobiological deficits seen in humans who suffer from PTSD (Aspesi and Pinna, 2019). The hypothalamic-pituitary-adrenal (HPA) axis-induced response to stressful stimuli, resulting in high cortisol levels, has been notoriously associated with the development of depressive symptoms (Wolkowitz et al., 2001; Almeida et al., 2021). Downregulation of neurosteroid biosynthesis, including the progesterone-derived and potent positive GABAergic modulator, allopregnanolone, has also been associated with PTSD symptoms both in male and female subjects (Rasmusson et al., 2006; Pineles et al., 2018; Rasmusson et al., 2019).

At the cellular level, PTSD has been associated with dysregulated neurotransmitter signaling and inflammatory response (Dong et al., 2020; Sun et al., 2021). This is related to an imbalance in reactive oxygen species (ROS) homeostasis, which is mainly regulated by mitochondrial function, thereby suggesting a link between oxidative impairment in mitochondrial function in response to chronic stress and mood instability (Lushchak et al., 2023). The ambivalent role of ROS in biological systems both hinders and provides support for strategies aimed at manipulating ROS levels for PTSD prevention. Increased ROS concentrations in PTSD have detrimental effects on calcium homeostasis, resulting in mitochondrial enlargement and leakage of Ca^2+^ ions, thereby creating a vicious cycle in which ROS enhance cytosolic Ca^2+^ levels, and Ca^2+^ enhances ROS production, which may worsen PTSD symptoms. Mechanisms by which monoamines, such as epinephrine (EPI), increase oxidative stress intensity following extracellular application include enhanced ATP production via glycolysis and oxidative phosphorylation. Stimulation of energy transformation by EPI in the cell increases flux of the intermediates of glycolysis and tricarboxylic acid cycle (TCA cycle). Side products of glycolysis such as methyglyoxal may induce carbonyl stress followed by oxidative stress due to increased ROS generation. Increased flux in TCA cycle in the mitochondria enhances production of reductive equivalents and flux of the electrons in the electron transport chain (ETC). Some of these electrons escape, ETC, to join molecular oxygen with formation of ROS, which may lead to intensification of oxidative stress. Oxidative damage of mitochondria may enhance inflammation by the release of damaged mitochondrial components into extracellular vesicles (EVs) (Todkar et al., 2021) or activation of the NRLP3 inflammasome (Zhou et al., 2011; Dong et al., 2020).

In this review, we discuss how mitochondrial dysfunction contributes to the manifestation of PTSD symptoms and how this may even increase PTSD susceptibility. Specifically, we review the mitochondrial involvement in the enhancement of ROS levels, targeting of EVs containing ROS-induced oxidized mitochondrial components, and the aggravation of oxidative and inflammatory stress in PTSD pathophysiology. In doing this, we explore the various potential treatment modalities through the regulation of these systems. We also address mitochondria-derived peptides (MDPs), which have been shown to counteract oxidative stress-induced mitochondrial dysfunction (e.g., Merry et al., 2020) as well as mitochondria-derived vesicles (MDVs), which play an important role in neuroprotective mechanisms (Hayakawa et al., 2016). Furthermore, we suggest a potential therapeutic approach to prevent or treat PTSD symptoms via interacting with MDVs content targeting or using MPDs, antioxidants and monoamine oxidase inhibitors.

## 2 Mitochondrial induction of oxidative stress in PTSD

Mitochondria are believed to be the main ROS producer in most cells. Because PTSD pathophysiology is so closely related to ROS homeostasis, it is important to analyze the processes related to ROS production, specifically within the mitochondria (Lushchak et al., 2023). In PTSD pathophysiology, enhanced ROS level can cause cell death via apoptosis. In this scenario, mitochondria using ROS can mediate apoptosis via cytochrome c release and caspase activation (Jia et al., 2018).

### 2.1 Activation of energy transformation processes

**FIGURE 1:**
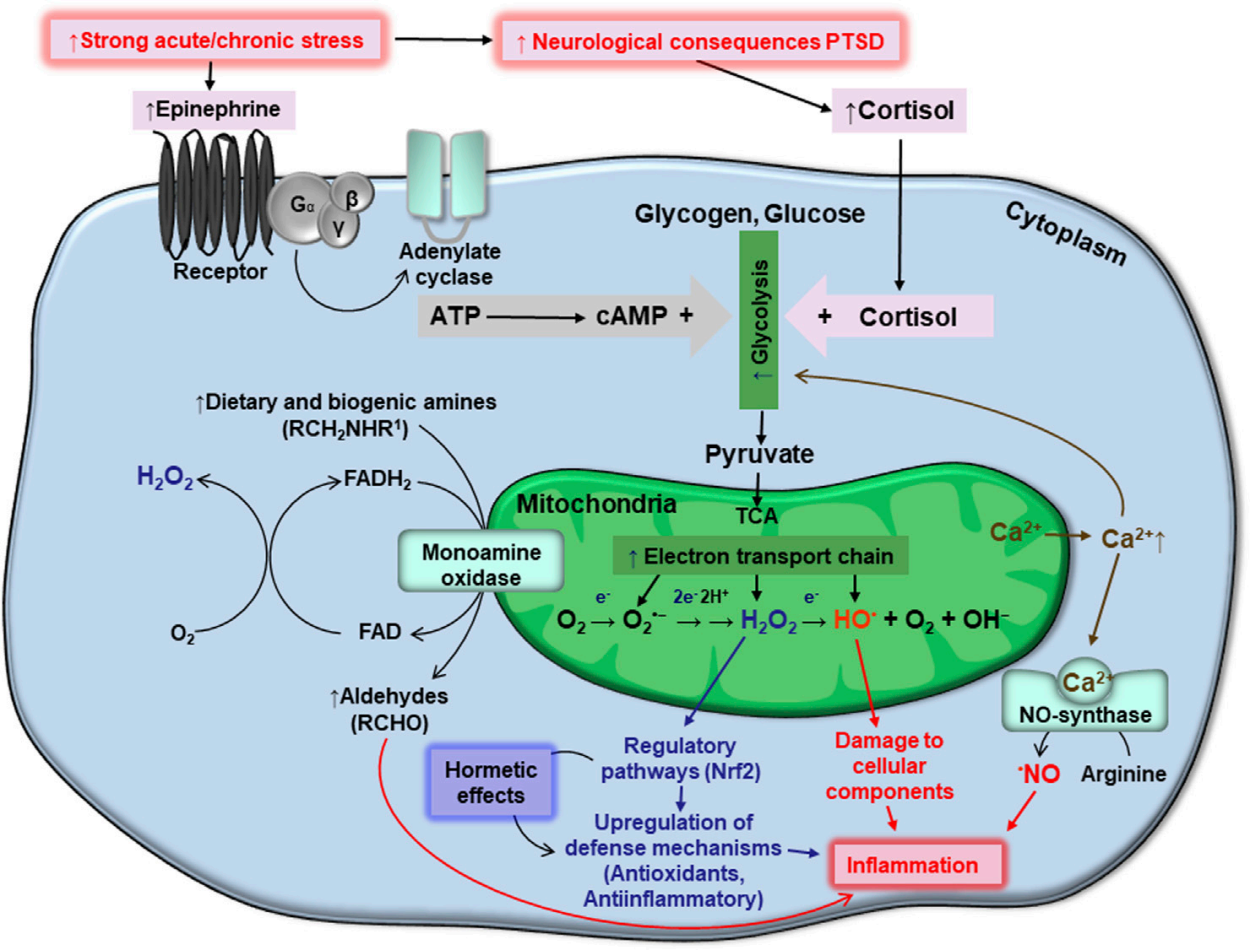
Schematic interplay between stresses, generation of ATP and reactive oxygen species, homeostasis of monoamines and calcium, oxidative stress, and PTSD. Details are provided in the text.

A single, or repeated, bout of acute stress which leads to development of PTSD may cause oxidative stress in humans and animals. During these stress events, organisms respond within the frame of classic stress reaction schemes, typically involving increased levels of stress hormones, primarily epinephrine (EPI, adrenaline) and cortisol, via activation of the hypothalamic-pituitary-adrenal (HPA) axis, which allows for the rapid mobilization of internal energy resources (McEwen et al., 2007) that produce the classic “fight or flight” response. Increases in EPI stimulate the adenylate cyclase cascade in target cells (Figure 1) including cellular receptors, G-protein coupled receptors, and protein kinase A. The latter phosphorylates phosphorylase kinase, which further phosphorylates low-active phosphorylase *b* converting it to highly active phosphorylase *a*. These events stimulate the rapid mobilization of glycogen reserves to fuel glycolysis. Starting with EPI binding to cellular receptors and glycogen phosphorolysis, the signal is believed to be amplified 10^9^ times. Phosphorolysis of glycogen provides higher levels of glucose-6-phosphate (G6P), an important intermediate in the glycolysis pathway (Popa et al., 2013). However, catabolic pathways at the G6P level are branched in a tissue/organ-specific manner. In the liver, where glycogen stores are highest, under stress conditions, a substantial part of formed G6P is dephosphorylated by glucose-6-phosphate phosphatase and released into the bloodstream to provide energy for muscle and brain tissue (Van Schaftingen and Gerin, 2002). In the liver and brain, some amount of G6P is also used by the pentose phosphate pathway (PPP), to provide NADPH for lipid biosynthesis, operation of antioxidant systems, and ribylose-5-phosphate for DNA reparation under stress conditions (Rajas et al., 2019). In the muscle, G6P is mainly used for glycolysis feeding mitochondria to release energy for muscle contraction (Jensen et al., 2011).

From an energetic point of view, glycolysis is not efficient; providing only two ATP molecules per glucose molecule used. Therefore, the end glycolytic intermediate, pyruvate, is used by mitochondria via the citric acid cycle in what is known also as the tricarboxylic acid cycle (TCA), or Krebs cycle, and the electron transport chain (ETC) to synthesize ATP. These systems are believed to generate 36 ATP molecules per glucose molecule used (Nelson and Cox, 2015).

It is important to note here, that during both glycolysis and the, ETC, deleterious side-products are formed. Some glycolytic intermediates are converted into dicarbonyls, such as glyoxal and methylglyoxal (Turk, 2010). These compounds will not be covered here.

In the mitochondria, more precisely in the ETC, some electrons escape at the level of complex I and III, and may interact with molecular oxygen to give rise to superoxide anions (O_2_^•−^) (Turrens, 2003). Spontaneously or enzymatically, due to the presence of mitochondrial matrix Mn-dependent superoxide dismutase (Mn-SOD), Cu-Zn-dependent superoxide dismutase (Cu,Zn-SOD) in the intermembrane space, O_2_^•−^ may be converted into hydrogen peroxide (H_2_O_2_), due to acceptance of an electron and two protons. Further, H_2_O_2_ may accept an additional electron and be converted into a hydroxyl radical (HO^•^) and hydroxyl anion (OH^−^). Finally, HO^•^ may accept one more electron and proton to give rise to a water molecule. Because O_2_^•−^, H_2_O_2_, and HO^•^, are more active than molecular oxygen, they are called reactive oxygen species (ROS). More specifically, O_2_^•−^ and HO^•^ are also considered free radicals, however, H_2_O_2_ is not. The chemical activity of these ROS from greatest to least is as follows: HO^•^ > O_2_^•−^ > H_2_O_2_. The hydroxyl radical is believed to be an especially damaging species, whereas H_2_O_2_ is frequently considered a signaling molecule (Lushchak, 2015). The superoxide anion radical is usually involved in both damaging and regulatory processes. Being a relatively inactive, small, and electroneutral molecule, H_2_O_2_ can readily cross biological membranes, allowing it to travel impressive distances (Mellon et al., 2019).

The question here is: which mechanisms are responsible for the suppression of glycolysis in PTSD? The inactivation of glycolytic enzymes by side-products of glycolysis and the ETC, namely, dicarbonyls and ROS, may be one of the reasons for that. It is well established that most of the known glycolytic enzymes are modifiable by these species, with the highest modification potential being for phosphofructokinase (PPK) and glyceraldehyde-3-phosphate dehydrogenase (GA3PDH) (Aggarwal et al., 2019). The first, PPK, is a key glycolytic enzyme believed to be responsible for more than 90% of all glycolytic flux (Reijenga et al., 2002). Inactivation of GA3PDH is important because of its potential to increase the production of a side-product, methylglyoxal, and redirect glycolytic intermediates to the biosynthesis of lipids. This crucial pathway may partially explain the connection between PTSD, overweight, obesity, and diabetes (Miller-Archie et al., 2014; Scherrer et al., 2018).

Increased utility of the TCA and ETC is important for efficient ATP production. However, this activation also results in increased ROS generation as side products. This may lead to the induction of oxidative stress. Based on intensity and duration, the stress may be either acute or chronic (Lushchak, 2011; Lushchak, 2015). Acute stress involves a single, short-term, event that triggers the stress response, leading to short-term oxidative stress that is usually resolved relatively soon after the stressor is lifted. Chronic oxidative stress may result from either a single bout of intense stress or repeated, chronic stress exposures. Here, we postulate a close association between chronic oxidative stress and PTSD.

### 2.2 Operation of the monoamine oxidase system

Above we highlighted the mechanisms by which extracellularly applied EPI may induce oxidative stress via activation of ATP production at the level of glycolysis and by oxidative phosphorylation. Several amines, including EPI, are involved in the cellular milieu. They are catabolized by the enzyme monoamine oxidase (MAO), which catalyzes the oxidative deamination of amines, which are further converted to aldehydes (Figure 1). The enzyme is attached to external mitochondrial membranes, and deaminates EPI, norepinephrine, dopamine, and several other dietary amines. During oxidative deamination, electrons from these substrates are used to reduce flavin adenine dinucleotide (FAD) to FADH_2_. The reduced coenzyme may then pass two electrons to molecular oxygen, giving rise to H_2_O_2_ (Di Sante et al., 2023). This provides a substantial pool of cytosolic H_2_O_2_.

### 2.3 Effects of cellular hydrogen peroxide

Hydrogen peroxide is a small uncharged molecule. H_2_O_2_ formed in the mitochondria readily leave this cellular compartment to the cytosol where it causes its effects, including direct oxidation of certain compounds. However, in accepting an additional electron, it is converted into the highly reactive ROS, HO^•^. The latter of which can “attack” any compound near its production. Such events modify chemical structure of the attacked molecules which results in change of functional properties such as inactivation. Fortunately, there are many systems to prevent HO^•^ formation, such as low molecular weight antioxidants like tripeptide glutathione, as well as various high molecular mass antioxidants. The best described are antioxidant enzymes, including SODs, catalase, and many peroxidases. By eliminating H_2_O_2_, whole antioxidant systems can prevent a deleterious scenario involving the formation of damaging HO^•^.

Besides the damaging effects of H_2_O_2_, specifically that of HO^•^, H_2_O_2_ can act as a signaling molecule (Sies, 2014). It is involved in the regulation of several pathways, but we will highlight the one most clearly related to PTSD; the KEAP1/Nrf2 system. Because PTSD leads to the development of oxidative stress (Miller and Sadeh, 2014; Oroian et al., 2021) coping with it cannot be overstated. ROS-mediated oxidation of certain cysteine residues of the cytosolic protein, KEAP1, results in increased levels of Nrf2. Nrf2 then localizes to the nucleus where it upregulates the expression of many genes encoding antioxidant enzymes as well as anti-inflammatory system components (Lushchak, 2015; Bayliak et al., 2023).

Herein lies the dualistic role of ROS in biological systems: both damaging and protective (regulatory). The approach for using ROS in PTSD prevention and its sequelae may involve the stimulation of defensive mechanisms. PTSD is associated with oxidative stress and enhanced ROS levels and therefore there is no reason to increase ROS production simply to upregulate the KEAP1/Nrf2 system. However, there are numerous modalities to stimulate the KEAP1/Nrf2 system pharmacologically. This is possible due to the KEAP1/Nrf2 system’s ability to be activated by ROS via oxidation of specific cysteine residues of KEAP1 protein. In addition to activation of the KEAP1/Nrf2 system by oxidation of KEAP1 cysteine residues, some cysteine residues of KEAP1 can interact with certain electrophilic compounds. This also prevents Nrf2 degradation and results in translocation of Nrf2 into the nucleus and upregulation of target genes (Hayes and Dinkova-Kostova, 2014; Dinkova-Kostova et al., 2018). There are a number of activators and inhibitors of the system with potential clinical significance (Robledinos-Antón et al., 2019). Sulforaphane is one of the activators of the KEAP1/Nrf2 system. Application of KEAP1/Nrf2 system activators may be used in PTSD, and can even be preferable over the use of classic antioxidants because they mobilize internal mechanisms. Nrf2 also upregulates the expression of antioxidant enzymes and expression of genes responsible for the enzymatic biosynthesis of glutathione, a particularly key enzyme of this pathway being glutamyl-cysteine ligase (Lushchak, 2012). Activators, as well as inhibitors, of the KEAP1/Nrf2 system have the potential to be used as part of a treatment approach for PTSD.

### 2.4 Calcium ions roles

Increased levels of ROS in PTSD also affect the homeostasis of calcium ions. Alterations in calcium homeostasis can lead to mitochondrial swelling (Müller et al., 2011), resulting in the leakage of Ca^2+^ ions from mitochondria. Importantly, Ca^2+^ is able to enhance ROS production (Madreiter-Sokolowski et al., 2020). Ca^2+^ effects are frequently mediated indirectly via Ca-binding proteins. The multifunction protein, calmodulin, is one of the best-studied Ca-binding proteins. It is a component of many multi-subunit oligomers, several of which may be directly implicated in PTSD. Complexed Ca-calmodulin, as a component of nitric oxide synthase (NOS), enhances the production of nitric oxide (^•^NO) (Schmidt et al., 1992). Similarly to H_2_O_2_, ^•^NO plays a dual role in biological systems. As a free radical, it can modify numerous cellular components, as described above (Lushchak and Lushchak, 2021). Additionally, ^•^NO may bind to soluble guanylate cyclase (SGC), which, when activated, regulates the contraction of smooth muscle. This may hold significance for PTSD states due to ^•^NO’s ability to induce vasodilation, thus improving blood supply to various organs, particularly the brain.

Calcium ions also have a dual effect on adenylate cyclase. First, Ca-calmodulin activates the phosphodiesterase of cAMP (and cGMP), reducing the levels of cAMP, and counteracting the stimulatory effects of extracellular EPI. Second, the opposite Ca^2+^ effect is related to its capability to complex with calmodulin to activate a component of adenylyl cyclase cascade, phosphorylase kinase. This acts synergistically with activated protein kinase A, which activates phosphorylase kinase via phosphorylation; whereas Ca-calmodulin directly stimulates phosphorylase kinase (Tokumitsu and Sakagami, 2022). It is important to note that Ca^2+^ also affects the production of reactive oxygen and nitrogen species and that these nitrogen species are also able to affect calcium homeostasis.

Ions of calcium may also directly coordinate regulation of the ATP supply and demand in vertebrates. At least, four mitochondrial dehydrogenases are activated by calcium ions (Denton, 2009). One of them, FAD-glycerol phosphate dehydrogenase is located on the outer surface of the inner mitochondrial membrane and is influenced by changes in cytoplasmic calcium ion concentration. The rest three Ca-sensitive enzymes, namely, pyruvate dehydrogenase, NAD-isocitrate dehydrogenase and oxoglutarate dehydrogenase are located within the mitochondria and are regulated by changes in concentrations of mitochondrial matrix calcium ions. Collectively, increase in calcium concentrations in cytosol and mitochondrial matrix enhance ATP production (Denton, 2009).

As mentioned above, ROS promote Ca^2+^ release from the mitochondria (Hamilton et al., 2018). They also inactivate Ca-ATPases in the endoplasmic reticulum and plasma membranes, Ca-channels, and inositol-1,4,5 triphosphate regulation of calcium homeostasis (Gutiérrez-Martín et al., 2004). Collectively, these ROS-based disturbances of calcium homeostasis increase levels of cytosolic Ca^2+^, forming a vicious cycle: ROS enhance cytosolic concentrations of Ca^2+^, and Ca^2+^enhance ROS (and RNS) generation. We postulate that this cycle may be overactive in PTSD. It is worthy to note that activation of adrenergic receptors may induce massive Ca^2+^ release from endoplasmic reticulum which can be important for coordination of cellular responses to stimulation (Motiejunaite et al., 2021).

### 2.5 ROS-related approaches to cope with PTSD

In this section we will build on the involvement of mitochondria in enhanced ROS levels and induction of oxidative stress in PTSD, and describe a molecular basis at least for two groups of treatments. The two modalities we will describe are 1) the usage of antioxidants, and 2) the usage of monoamine oxidase inhibitors. Since PTSD is well-established to be associated with oxidative stress (Miller et al., 2018; Li W et al., 2020; Peruzzolo et al., 2022; Lushchak et al., 2023)), this provides support for the theory of decreasing oxidative stress levels down to a basic steady-state in order to treat PTSD (Lushchak, 2015). This strategy may be accomplished in two ways: 1) use of dietary antioxidant sources or food supplements either as food components or special additives, and 2) activation of endogenous antioxidant systems. Natural antioxidants can be found in fruits and vegetables. They include carotenoids and anthocyanines, among others. Animal-derived foods may also be helpful in providing a “primitive” source of ideal proteins, vitamin A, and other antioxidants. They can be used as precursors for biosynthesis of peptides. Specific products, such as milk whey, may be a good source for biosynthesis of the powerful antioxidant, glutathione (Lushchak, 2012). The second approach consists of inducers of cellular antioxidant systems, such as activators of the KEAP1/Nrf2 system. Due to the presence of oxidative stress in PTSD, additional activation by ROS is nonsensical. Pharmacologic activation via electrophiles on the other hand, may hold promise. However, this would require a detailed investigation into the effects of potential overactivation of the KEAP1/Nrf2 system. For example, activation of drug-metabolizing enzymes is a risk to consider, as previously described (Wu et al., 2012). In practice, concerted utilization of both of the described approaches would be preferential. Importantly, the beneficial effects of antioxidants in PTSD were reported (Solanki et al., 2015; Kaplan et al., 2018; de Souza et al., 2019), whereas pharmacologic activation of the KEAP1/Nrf2 system in PTSD, and other neurological pathologies, awaits further investigation.

What is the mechanism underlaying MAO inhibitors’ suppression of ROS production? They can just inhibit the enzyme (Behl et al., 2021)! This inhibition of MAO decreases H_2_O_2_ production and the deleterious effects of ROS in PTSD. However, this results in higher intracellular levels of biogenic amines, which to some extent may be beneficial, but at high levels, this increase may have negative consequences. For example, EPI may enter autoxidation due to ROS generation (Álvarez-Diduk and Galano, 2015). Importantly, MAO inhibitors may be utilized to treat PTSD (Zohar et al., 2000; Hunter et al., 2022). But in practice, only inhibitors of serotonin reuptake are applied.

## 3 Role of mitochondria in preventing/treatment of oxidative stress in PTSD

Available data indicate that mitochondrial dysfunction, or suboptimal functioning, inherited or acquired, contribute to the manifestation of PTSD symptoms and can increase susceptibility to PTSD (for review, see, e.g., Preston et al., 2018; Mellon et al., 2018; Kaplan et al., 2023; Sumner et al., 2023). Reduction of oxidative stress appears to be a reasonable therapeutic approach in the treatment or prevention of PTSD. This can be achieved by supporting mitochondrial function via endogenous antioxidant pathways. Increasing knowledge on mitochondria has the ability to elucidate new factors of putative medical significance and therapeutic potential.

### 3.1 Mitochondria-derived peptides

Mitochondria-derived peptides (MDPs), also known as mitochondrial microproteins, are encoded by short open reading frames (ORFs) located in mitochondrial DNA (mtDNA) (e.g., Kim et al., 2017; Merry et al., 2020; Miller et al., 2022). Silico analysis indicates the presence of about 400 putative MDPs in the human mitochondria, containing anywhere from 9 to 40 amino acids (Miller et al., 2020), although as of today, only eight MDPs have been confirmed experimentally. They include: 1) humanin, encoded by the 16S rRNA region of mtDNA (Hashimoto et al., 2001); 2) Mitochondrial ORF of the 12S rRNA Type-C (MOTS-c), encoded by the 12S rRNA region of mtDNA (Lee et al., 2015) and 3) six small humanin-like peptides (SHLPs 1–6), also encoded by the 16S rRNA region (Cobb et al., 2016). Importantly, these MDPs are known to counteract oxidative-stress-induced mitochondrial dysfunction (e.g., Merry et al., 2020).

According to PubMed, the most well-studied of these MDPs is humanin. Data on different cell types indicate that this MDP attenuates ROS-mediated oxidative damage (e.g., Gong et al., 2018; Cai et al., 2021). Humanin is known to influence the mitochondrial antioxidant defense systems by increasing levels of Cu, Zn-superoxide dismutase (SOD1), mitochondrial glutathione, and by modulating the mitochondrial respiratory chain (Klein et al., 2013; Matsunaga et al., 2016; Thummasorn et al., 2018; Zhao et al., 2021). It has been also shown to upregulate mitochondrial transcription factor A (TFAM) (Sreekumar et al., 2016), known to play a crucial role in stabilizing mtDNA through the formation of nucleoids, which are important to counteract mtDNA release (Decout et al., 2021). Additionally, humanin is known to downregulate the expression of a gene encoding thioredoxin interacting protein (TxNIP), and decrease formation of carbonyl groups (Li F et al., 2020), which serve as markers of oxidative stress (e.g., Anderson, 2016). Interestingly, humanin has been found to exert a protective role, especially in nervous tissue (Lei and Rao, 2022). However, humanin has also been shown to enhance progression of Triple Negative Breast Cancer (Ayala et al., 2020), which should be taken into account when considering it for PTSD pharmacotherapy.

The ability to attenuate oxidative stress and the subsequent inflammatory response has been also reported for MOTS-c (e.g., Shen et al., 2022), the only MDP commercially available. MOTS-c is known to stimulate AMP-activated protein kinase (AMPK) (Lee et al., 2015), described as a guardian of metabolism and mitochondrial homeostasis (Herzig and Shaw, 2018). Moreover, it has been shown that oxidative stress can induce rapid translocation of MOTS-c to the nucleus in an AMPK-dependent manner, which indicates the peptide’s important role in regulating expression of nuclear genes, allowing for resilience to stress-states (Kim et al., 2018).

The dual mitochondrial/nuclear localization is also shown for SHLP 2 and 3, whereas SHLP 1, 4, 5, and 6 are located exclusively in the mitochondria (Cobb et al., 2016). SHLP1-6 differ in their expression patterns and their ability to regulate mitochondrial function. Similar to humanin, both SHLP2 and SHLP3 were shown to protect against oxidative-stress-mediated mitochondrial dysfunction, and enhance neural cell survival, but further work is needed to elucidate the specific mechanisms behind SHLPs’ effects (Cobb et al., 2016; Miller et al., 2022). Nevertheless, it is worth mentioning that SHLP2 has been shown to have a protective effect in neurodegenerative diseases for its ability to prevent mitochondrial loss (Thiankhaw et al., 2022).

MDPs are known to act as both paracrine and endocrine signaling molecules (e.g., Merry et al., 2020), and have emerged as promising biomarkers, as well as potential therapeutic targets to treat neurodegenerative, cardiovascular, and metabolic conditions (e.g., Miller et al., 2022; Thiankhaw et al., 2022). However as of yet, MDPs have not been studied in the context of PTSD. Nevertheless, their antioxidant activity within mitochondria, and resulting cytoprotective effect, provides a promising starting point for elucidating their role in the pathophysiology of PTSD, and the development of putative prevention and treatment strategies. One important consideration for further study, however, is the issue of single nucleotide polymorphisms (SNPs) affecting MDP sequence and functionality. One SNP, rs2854128, for example, in the mtDNA coding region for humanin, is associated with accelerated cognitive decline during aging, supporting the assumption that this MDP variant actually has neurodamaging, rather than protective effects (Yen et al., 2018). The issue of possible MDP variants is excellently discussed by Miller et al. (2022). The review describes known SNPs in MDPs encoding sequences and their association with human diseases including neurodegenerative ones. It also focuses on the importance of the applied methodology for analysis of SNPs occurring in mtDNA.

### 3.2 Mitochondria-derived vesicles

It is well known that cells secrete mitochondrial proteins, mtDNA, and even intact mitochondria as part of mitochondrial quality control (e.g., McLelland et al., 2014; Sugiura et al., 2014; Heyn et al., 2023), long-range metabolic regulation (e.g., Hough et al., 2018; Heyn et al., 2023) and stimulation of the immune response (e.g., Zhou et al., 2011; Puhm et al., 2019). Available data indicate that the process of secretion is mediated by extracellular vesicles (EV), and requires first the formation of mitochondria-derived vesicles (MDVs), which is regarded as a conserved phenomenon (e.g., Todkar et al., 2021; Vasam et al., 2021; Heyn et al., 2023). MDVs emerge as an essential quality control mechanism in response to mitochondrial stress, in addition to mitophagy and fission/fusion processes that regulate mitochondrial turnover (Heyn et al., 2023). Accordingly, the formation of MDVs is enhanced by oxidative stress (Cadete et al., 2016). Moreover, the content of MDVs may be altered by oxidative stress (Vasam et al., 2021). Consequently, it is thought that MDVs represent a cellular oxidative stress response that may contribute to different disease etiologies (Heyn et al., 2023).

Upon oxidative mitochondrial damage, a greater number of MDVs are targeted to lysosomes for degradation, which in turn blocks the secretion of mitochondrial content by EVs (Todkar et al., 2021). The formation of ROS-induced, MDV-containing, oxidized mitochondrial components is known to be stimulated by the PINK1/Parkin pathway (McLelland et al., 2014). Interestingly, a genome-wide association study of PTSD has indicated the Parkin gene as a significant locus in the context of PTSD (Nievergelt et al., 2019). Moreover, available data indicate that brain cells produce MDVs and EVs (D'Acunzo et al., 2021). It is assumed that these vesicles may be part of a neuroprotective mechanism consisting of the transferring of “repair packages” from one cell to another (Hayakawa et al., 2016; D'Acunzo et al., 2021). However, when the release of damaged mitochondrial components into EVs is not blocked, immune activation may occur (Todkar et al., 2021). Accordingly, neuron- and astrocyte-derived EVs recently isolated from the plasma of PTSD patients were shown to affect astrocyte-neuron communication in PTSD, but the relationship of this phenomenon with mitochondria was not studied (Guo et al., 2023).

It appears that MDVs and EVs could be addressed clinically, although more research is required to fully use the diagnostic and therapeutic potential of MDVs. This is particularly relevant to PTSD as it seems the detection of MDV formation, and analysis of their contents particularly after secretion, could be an efficient diagnostic approach. Moreover, delivery of functional mitochondrial proteins to repair organelle function, or to modify MDV content, can also be considered as a possible therapeutic strategy.

## 4 Conclusion and perspectives

Application of antioxidants, pharmacologic activators of endogenous antioxidant systems, selective serotonin reuptake inhibitors, and MAO inhibitors may serve to decrease the deleterious effects of ROS in the pathogenesis of PTSD. However, some caution must still be used. Excessive suppression of ROS production and elimination may have negative functional effects on the organism, decreasing its signaling ability, or weakening its defense mechanisms. Ultimately, the proper balance between ROS formation and elimination has to be maintained. To accomplish this, it will require monitoring of ROS homeostasis. So, the development of a reliable noninvasive, or minimally invasive, system for monitoring the balance of pro-oxidative and anti-oxidative processes in PTSD is needed. This allows for a direct avenue to tightly control oxidative stress in PTSD, as well as other oxidative-stress related pathologies. As we have outlined here, this can be achieved with the consideration of mitochondrial-mediated, endogenous antioxidative mechanisms, including both MDPs and MDVs.
